# *De novo* transcriptome dataset of *Stevia rebaudiana* accession MS007

**DOI:** 10.1016/j.dib.2019.104811

**Published:** 2019-11-15

**Authors:** Nurul H. Samsulrizal, Khairul S. Khadzran, Siti HN. Shaarani, Abdul L. Noh, Tamil CM. Sundram, Mohd Azrul Naim, Zarina Zainuddin

**Affiliations:** a Department of Plant Science, Kulliyyah of Science, International Islamic University Malaysia, Jln. Sultan Ahmad Shah, Kuantan, Pahang, Malaysia; b Research Unit for Bioinformatics and Computational Biology (RUBIC), Kulliyyah of Science, International Islamic University Malaysia, Jln. Sultan Ahmad Shah, Kuantan, Pahang, Malaysia; c Faculty of Chemical & Natural Resources Engineering, Universiti Malaysia Pahang, Lebuhraya Tun Razak, Gambang, Pahang, Malaysia

**Keywords:** RNA-Seq, Transcriptome, *Stevia rebaudiana*, Flowering genes

## Abstract

*Stevia rebaudiana* (*S. rebaudiana*) is a herbaceous and perennial plant belonging to Asteraceae family. The genus stevia is well known as a natural producer of sweetener comprising non-caloric and non-carcinogenic steviol glycosides. In recent years, the capability in producing natural sweetner has increased the demand for *S. rebaudiana* as substitute of processed sugars. Flowering phase of *S. rebaudiana* has shown to affect the content of steviol glycosides in the leaves*.* Steviol glycosides level is the highest at the time of flower bud formation and lowest at time preceding and following flower bud formation. Therefore, sequencing and analysing the genes that are involved in flowering phase will provide platform for gene manipulation in increasing steviol glycosides content. The Stevia transcriptome data that include two stages of growth (before flowering and after flowering), were obtained using Illumina RNA-seq technology and can be accessed at NCBI Sequence Read Archive under Accession No. SRX6362785 and SRX6362784.

**Table:** Specifications

Subject	Agricultural and Biological Sciences
Specific subject area	Plant Science
Type of data	Transcriptome data
How data were acquired	Paired end transcriptome of *Stevia rebaudiana* was sequenced using Illumina at 1st Base Laboratory Sdn Bhd (Malaysia)*. De novo* transcriptome assembly was performed by using Trinity RNA-Seq v2.4.0.
Data format	Raw sequences (FASTQ)
Experimental factors	Samples were collected at two different stages of plant growth, (before and after flowering of Stevia).
Experimental features	*S. rebaudiana* leaf samples were collected from plants grown in Glasshouse and Nursery Complex (GNC) IIUM Kuantan Campus.
Data source location	Kuantan, Pahang, Malaysia.
Data accessibility	Raw FASTQ files were deposited in NCBI SRA database with the accession number SRX6362785 (https://www.ncbi.nlm.nih.gov/sra/SRX6362785) and SRX6362784 (https://www.ncbi.nlm.nih.gov/sra/SRX6362784)

**Table:** 

**Value of the Data** • The data obtained using Illumina sequencer is the first source of S. rebaudiana RNA-seq • The data provide a glimpse into molecular perspectives of S. rebaudiana and contribute further to gene manipulation for interest experiment. • The data presented here can be used for genes discovery of processes involved in flowering, that affect the content of steviol glycosides. • The data also can used in unravelling genes and pathways involved in the biosynthesis of steviol glycosides.

## 1 Data

The dataset contains raw sequencing data obtained through the transcriptome sequencing of leaf samples of *S. rebaudiana* (accession number MS007) collected at two different stages of plant growth, (i) 1 week before flowering and (ii) after flowering. The plant was grown at Glasshouse and Nursery Complex in IIUM Kuantan Campus. The bam files that obtained from transcriptome libraries have been deposited to NCBI SRA database with accession number SRX6362784 and SRX6362785. The statistic of RNA-seq generated and overview of the number of transcripts are described in Table 1, Table 2, respectively.

**Table 1:** Statistic RNA-seq generated from two different libraries.

Sample	Before flowering Raw Reads	After flowering Clean reads
Raw Reads	62737178	53452080
Clean reads	44277294	47676660
Clean bases	7.2G	6.6G
GC (%)	45.06	45.24

**Table 2:** Overview of the number of transcripts from assembly using Trinity RNA-Seq v2.4.0.

Attributes	Transcripts	Unigenes
Min Length	201	201
Mean Length	976	976
Median Length	618	618
Max Length	131182	131182
N50	1380	1380
Total Nucleotides	105718330	105717560

## 2 Experimental design, materials, and methods

### 2.1 Sample preparation

*S. rebaudiana* accession MS007 was cultivated through shoot cutting method, in order to grow sufficient number of plants required. A healthy *S. rebaudiana* accession MS007 was obtained from Glasshouse and Nursery Complex in IIUM, Kuantan Campus as a mother plant. Healthy shoot with at least 2 leaves were selected for shoot propagation [1]. No additional chemical or organic fertilizer was added and the plants were allowed to grow for 40 days until flowering [2].

### 2.2 RNA isolation and library preparation

RNA was isolated from the samples using Plant Total RNA Mini Kit (Geneaid). Transcriptome data were generated from the total RNA extracted from these two samples collected at two different developmental stages. NanoDrop ND-1000 Spectrophotometer (NanoDrop Technologies, USA) and Bioanalyzer RNA 6000 chips system (Agilent Technologies, USA) were used to determine the quality and integrity of total RNA.

### 2.3 RNA-seq data analysis

The raw reads generated from *S. rebaudiana* samples were trimmed using Solexa QA++ with Phred score Q20. By using FastQ file, the FastQC was ran at default parameter. *De novo* assembly of *S. rebaudiana* data was done using Trinity RNA-Seq 2.0 with default settings [3]. The details of sequencing and assembly data is given in Table 1, Table 2.
